# Data set for transcriptome analysis of the Chinese giant salamander (*Andrias davidianus* )

**DOI:** 10.1016/j.dib.2015.11.042

**Published:** 2015-11-25

**Authors:** Xuemei Jiang, Yuan Wang, Xiaoying Zhang

**Affiliations:** College of Veterinary Medicine, Northwest A&F University, Yangling, Shaanxi, China

**Keywords:** Andrias davidianus, Transcriptome

## Abstract

The Chinese giant salamander (*Andrias davidianus*) occupies a seat at the phylogenetic and species evolution process, which makes it an invaluable model for genetics; however, the genetic information and gene sequences about the Chinese giant salamander in public databases are scanty. Hence, we aimed to perform transcriptome analysis with the help of high-throughput sequencing. In this data, 61,317,940 raw reads were acquired from Chinese giant salamander mRNA using Illumina paired-end sequencing platform. After de novo assembly, a total of 72,072 unigenes were gained, in which 33,834 (46.95%) and 29,479 (40.91%) transcripts exhibited homology to sequences in the Nr database and Swiss-Prot database, (*E*-value <10^−5^), respectively. In the obtained unigenes, 18,019 (25%) transcripts were assigned with at least one Gene Ontology term, of which 1218 (6.8%) transcripts were assigned to immune system processes. In addition, a total of 17,572 assembled sequences were assigned into 241 predicted KEGG metabolic pathways. Among these, 2552 (14.5%) transcripts were assigned to the immune system relevant pathway and 5 transcripts were identified as potential antimicrobial peptides (AMPs).

**Specifications Table**

**Table:** 

Subject area	Biology
More specific subject area	Bioinformatics and immunology
Type of data	transcriptome
How data was acquired	high-throughput RNA-sequencing using an in-house workflow of Novogene
Data format	analyzed
Experimental factors	RNA Isolation, cDNA library construction and sequencing
Experimental features	Transcriptome analysis of Andrias davidianus
Data source location	Yangling, Shaanxi, China
Data accessibility	The data is available with this article

**Value of the data**

• *Andrias davidianus* occupies a seat at the phylogenetic and species evolution process, which makes it an invaluable model for genetics.
• The genetic information and gene sequences about the *A. davidianus* in public databases are scanty.
• It is a significant contribution to the further research of genetic immunology of amphibians.

## 1 Data

See Supplementary Table 1

## 2 Experimental design, materials and methods

### 2.1 Animal material and RNA Isolation

Two healthy Chinese giant salamanders of two ages (one-year-old and two year-old) were collected from a farm in HanZhong, Shaanxi Province, China. Total RNA was isolated from the spleen tissues using RNAsimple Total RNA Kit (Tiangen Technologies Inc., Beijing, China). And the RNA integrity score and quantity was checked by RNA 6000 Nano Assay Kit with a Bioanalyzer 2100 (Agilent Technologies) prior to cDNA synthesis.

### 2.2 cDNA library construction and sequencing

Beads with oligo-dT were used to isolate poly-A mRNA after total RNA purification. The mRNA was disrupted into short fragments with fragmentation buffer [1]. These short fragments can serve as templates to synthesize first-strand cDNA by using random primers. Then the second-strand cDNA was synthesized using RNase H and DNA polymerase I [1]. The short fragments were linked to sequencing adapters and then screened as templates by electrophoresis for PCR amplification [2]. The cDNA library was sequenced on an Illumina HiSeq2000 platform.

### 2.3 Data filtering and de novo assembly

Low quality sequences (<Q20) was removed using an in-house workflow of Novogene before the de novo assembly. Then, the remained reads were assembled using Trinity with a strategy of multiple K-mer lengths and coverage cut-off values [3], [4].

### 2.4 Gene Annotations and Classifications

All unigenes sequences were used to make sequence alignment with the NCBI non-redundant nucleic acid database (NT), the NCBI non-redundant protein database (NR), the Swiss-Prot database, and the Clusters of Orthologous Groups database using BLASTx with an *E*-value less than 1e^−5^. The unigenes sequences were also aligned based on the KEGG database to analyse metabolic pathways [5], [6]. Functions categories were carried out based on the COG database [7], [8].

### 2.5 Molecular markers

A microsatellite program (MISA; http://pgrc.ipkgatersleben. de/misa/) was used to identify and localize microsatellite motifs [9].
