# Supplementary material for: Data set for transcriptome analysis of the Chinese giant salamander (Andrias davidianus )
Source: Data Brief. 2015 Nov 25;6:12–4. doi: 10.1016/j.dib.2015.11.042 (PMC4683342; doi:10.1016/j.dib.2015.11.042)
Supplement: Supplementary file 2 — Supplementary material [file mmc2.docx]

**Conflict of Interest**

The authors declare that there are no conflicts of interest. We confirm that the manuscript has been read and approved by all named authors and that there are no other persons who satisfied the criteria for authorship but are not listed. We further confirm that the order of authors listed in the manuscript has been approved by all of us.

**Corresponding author:**Dr. X.Y. Zhang, College of Veterinary Medicine, Northwest Agriculture and Forestry University, Yangling, 712100, China Tel./Fax: +86 29 8709 1239. E-mail address: [zhang.xy@nwsuaf.edu.cn](mailto:zhang.xy@nwsuaf.edu.cn).
